# The Christian-Science Bubble

**Published:** 1900-08

**Authors:** C. A. F. Lindorme

**Affiliations:** Chicago, Ill.


### THE CHRISTIAN-SCIENCE BUBBLE.

By C. A. F. LINDORME, Ph.D., M.D.,
Chicago, Ill.

The brazen-fronted impudence of this twentieth-century-style-resurrection of the ages of public crazes, the flagellating piousness, the religious dancing, etc., which a sanguine student of history would have thought incompatible with the awaking of the critical propensities of our epoch, is typically in the pennant which flies above the masthead of the bogus craft. Like all pirate outfits, she heels the suspicious port she hails from, and has spurious ship's papers to bolster mendacious record and relations. Science is called that knowledge which renders account of itself by establishing a title for every item in the demonstration or description of the nature of the connections averred. It is this *methodological* character by which science is distinguished from empiricism and all other loose talk, which cannot be brought in a system for want of such coherence of its different parts, and on account of the lack of all harmonious correspondence with the main tracks of intellectual work. There is nothing which constitutes a more precious human achievement than this methodological gain. If all the discoveries made in three hundred years were lost, all the inventions since that of movable type were taken from us, if the knowledge of the scientific method remained unimpaired there would be little harm done: all the inventions, all the discoveries would soon be renewed.

It is a fact that methodology is not an absolute preventive of cranks and craziness. Either of them will lower upon the profession in the midst of its coryphæs, even. We have seen Brown-Séquard defend the luminous theory to reestablish the lustiness of youth by feeding rabbit-testicles, and Metchnikoff comes now forward to tell us that he can beat Methuselah by fighting the deadly microbe. But such eccentricities are tolerated in the ranks of the profession only as incandescence of $CH_4$, which has nothing to do with the progress of enlightenment. The Christian-Science fustian places the glimmer of such will-o'-the-wisps at the head of all illumination. They propound that, in order to do well in medicine and surgery, the work must be given to one who understands least about it, and who poses as a superior original for no other reason but because of sitting by and doing nothing.

We admit this doing nothing is not all they do. They pray besides. But this praying is no practice of medicine, and still less of surgery. It is something, moreover, we can prove which defends itself from the standpoint of orthodox religiousness, especially with regard to medicine. There was a time, it is true, when the faithful of the civilized world believed in a personal reign over the cosmical environment. Likewise as the Indians were overawed by Columbus's forecast of the eclipse, it was believed by the Europeans that once a Jewish general, by invoking the supreme providential power, postponed the sunset, securing thereby the necessary light to whip his enemies—something the providential power was very partial about, just because it concerned his sweet Jews; and Bohemians, Poles, Greeks and Armenians, Italians and Spaniards may believe in it to-day, but scientific Christians do not. In orthodox theology, even, long ago the standpoint was given up to try to use the prayer as an instrument of technical world-rule. The prayer is by sensible theologians not considered any more as a means of objective influence, but only as one of subjective benefit. It is the fashion among Christian people, when they set about killing each other in battle, to pray for the victory. But the soldiers are well aware that they are not warranted in the reliance on it. The pitch by which it goes in a pitched battle is in a different pitcher than that which holds the holy water. But there are lots of soldiers who are braced up by the prayer. It rallies their metaphysical powers, and that is liable to make them stronger. The prayer is calculated to raise the warrior beyond himself, to make him forget and pass by the paltry cares of his individuality and reflect on the great interests he has enlisted for. Likewise an unfortunate exasperate in ills, who is driven to despair, can by prayer be relieved and given back to himself if he gathers strength in the solace of a higher order of things than that under which he broke down: he gains confidence that he is not abandoned altogether, but lovingly cared for by an imagined omnipotence. None the less a philosoper can derive the like solace without any prayer, from the consideration that the single individual is only the way in which nature exhibits, and that immortality, eternity and infinity are to be seen only in the great interest of the universe; that consequently it is these under which the interests, the cares of the man should be considered and estimated. But to think that technically the prayer realizes the result which it anticipatingly vociferates is a stultification of the intellect; and if the intelligence-bragging American, in saying his prayer, as I heard one the last time I was in church, yells at the top of his voice lest he incur the danger of not being fervent enough, he ranks no higher in civilization than the Lama of the Tartars, who have prayer-barrels driven by water-power, in order to turn out more piousness and render the praying thereby a more lucrative work.

The proceeding of the Christian-Science people is therefore not only medically criminal, because ignoring intentionally a real danger and patching it up with their arrogance, but also theologically stupid, because it is a relapse into an exploded theory long ago abandoned by all the authorities in the field.

There is, however, a point which, if it does not exonerate the Christian-Science humbugs, throws a side-light on medicine which, in the camera obscura of objectivation, turns out a noteworthy photograph. If the Christian-Science practice is a criminal absurdity and a most ignominious imposition, we must, with regard to it, bear in mind, for all that, that there is no social movement, howsoever perverse, but it is the exponent of a social need of some kind which wants to be attended to and the neglect of which is that which caused the irrational vicariousness of gratification. Just as the frequent lynching in the United States is a consequence of the forensic laxity of our judiciary, and as very frequently the hankering of children for candy is a consequence of their being stinted with sugar at their regular meals, so the metaphysical nonsense of the Christian-Science people is due to the psychological indifference in the medical profession. We may boast of enormous strides in all technical matters, our achievements in general surgery and the specialties are enormous; but we have left a sore and sadly cleaving gap on the field where physiology merges into psychology. The therapeutics of psychiatry have been worked up masterly, but show no genesis from their accompanying theory. While the former has been cleverly developed from careful observation, the latter was drowned in vocabulation, and its authors were more studious to make connection with the old metaphysics of scholastic extraction than with the inductive philosophy implicitly worked out of their own empiricism. And similarly in medicine: with the model of the surgical preferment before them, the mental relations in theory and practice have been by the profession slighted, all the clinical indications notwithstanding, as in the case of color-and-sound therapeutics, light and music being raised to the dignity of subjects of materia medica. Hence the exploitation by Christian Science, so-called. The public was by the regular medical counsel left without mind therapeutics, and consequently attended to its own supply, which, as a matter of course, was accordingly (*quam suis*).

The French have a proverb which says: "*Il est bon dans son genre, mais son genre n'est pas bon.*" (He is good in his way, but his way is no good.) This does not apply to the Christian-Science perpetrators. Not alone that the course they pursue is bad; they do it also in a most faulty style. Once a disease there, all psychological influence is of very little promise, although this little is in very many cases much more than by most practitioners is admitted. But as a preventive, especially in alienation of the mind, psychology is a remedy of most astounding effectiveness and proportions. And this refers not only to the pathology of individual cases, but to the mischief in that line which may be traced to the sins of public opinion, education and the like. There can be no doubt that in never so many cases public opinion, to all intent and purpose, is calculated to create an environment which is exceedingly favorable to the fostering of insanity. To the attentive and careful observer it must be clear that we have the *money-mania* as a new form of madness, and that the same is due to the neglect of preventive psychology. If, therefore, the medical profession thinks it urgent to take action in this Christian-Science affair, the negative aggression should not be omitted, which consists in a scientific taking up of the mental medication which is being so grossly sophisticated by charlatan adulteration.

